# Iron and Ferritin Levels in the Serum and Milk of Bovine Leukemia Virus-Infected Dairy Cows

**DOI:** 10.3389/fvets.2015.00012

**Published:** 2015-05-26

**Authors:** Star A. Schnell, Hiromichi Ohtsuka, Seiichi Kakinuma, Yasunaga Yoshikawa, Kiyotaka Watanabe, Koichi Orino

**Affiliations:** ^1^Laboratory of Veterinary Biochemistry, School of Veterinary Medicine, Kitasato University, Towada, Japan; ^2^Large Animal Clinical Sciences, School of Veterinary Medicine, Rakuno Gakuen University, Ebetsu, Japan; ^3^Kakinuma Veterinary Hospital, Honjou, Japan

**Keywords:** bovine leukemia virus, dairy cow, iron, milk, serum

## Abstract

Iron metabolism was examined in 15 bovine leukemia virus (BLV)-infected dairy cows (2.6–7.8 years old). BLV infection was detected by measuring serum antibody titer against BLV virus antigen (gp51). The anti-BLV antibody titers of the BLV-infected cows were significantly higher in serum than in milk; a single serum-positive animal lacked detectable anti-BLV antibodies in its milk. Iron and ferritin concentrations also were significantly higher in serum than in milk. Although most of the BLV-infected dairy cows had past or present anamneses (such as inflammatory diseases, including intramammary infection), the milk ferritin concentrations of the infected cows were significantly lower than those of normal cows; serum ferritin concentrations did not differ significantly between these two groups. The anti-BLV antibody titers in milk samples showed significant correlation with serum iron concentrations. These results suggest that BLV infection affects iron homeostasis through iron metabolism in the dairy cow mammary gland.

## Introduction

Bovine leukosis is classified into two types: sporadic bovine leukosis (SBL, characterized by T-cell leukosis) and enzootic bovine leukosis (EBL, characterized by B-cell leukosis) (1–5). EBL, which occurs at a much higher frequency than SBL, and is an infectious disease caused by bovine leukemia virus (BLV) (4). This virus belongs to the genus *Deltaretrovirus* in the family *Retreviridae* (1, 3). BLV infection affects cellular functions in transcription, translation, RNA processing, signal transduction, cell growth, apoptosis, stress response, and immune response (3, 6–12). BLV is a unique retrovirus related to human T-cell leukemia virus type 1 (HTLV-1), which is a causative agent of adult T-cell leukemia (1, 13). In the BLV and HTLV-1 systems, cell transformation may be the result of the expression of the Tax protein (5, 6, 13). Molecular understanding of the leukemogenesis induced by BLV infection may provide insights for the development of novel and effective antiretroviral therapeutics applicable for HTLV (1).

Bovine leukemia virus infection is frequently asymptomatic; in many cases, infected animals remain virus carriers for life without exhibiting signs of infection (1, 13). Approximately 30% of infected cattle develop persistent lymphocytosis (PL), which is characterized by permanent and relatively stable increases in the number of B lymphocytes in the peripheral blood while resulting in fatal leukemia or lymphoma in fewer than 5% of the infected cows (5, 6). BLV is transmitted horizontally through transfer of biological fluids contaminated with BLV-infected B-lymphocytes, such as inappropriate re-use of needles and gloves for rectal examination; through milk; and possibly via insect bites (1, 5). BLV-infected cows with subclinical mastitis may be sources of infectious transmission, since these animals produce milk that contains BLV-infected lymphocytes (14, 15).

Serum ferritin is the best indicator for estimating body iron stores, whereas serum ferritin provides a marker for malignant and inflammatory conditions (16). Significantly higher serum ferritin levels were observed in leukemic cows compared to normal animals (17). Although BLV-infected dairy cow may appear healthy, BLV infection may cause economic loss in cattle production and export (1, 3, 5, 18). Although the association between BLV infection and mastitis in dairy cows remains controversial (14, 15, 19), BLV infection is believed to induce mastitis due to the virus’ immunosuppressive effects (14). Additionally, serum iron levels are decreased in animals suffering from inflammatory diseases, an effect that results from IL-6 mediated hypoferremia (20). By contrast, serum ferritin can be an inflammatory marker of acute-phase infection, although elevated serum ferritin levels can lead to misdiagnosis of anemia (21). Furthermore, milk ferritin levels are increased in response to intramammary infection (22).

Despite this previous work in bovids, iron metabolism of BLV-infected dairy cows has not been studied. There is still controversial in the relation between BLV-infection and mastitis (14, 15, 19). Serum ferritin may be indicator of BLV infection stage as in tumor marker (23). The purpose of this study was to elucidate the effect of BLV infection on iron metabolism in dairy cows.

## Materials and Methods

### Chemicals

Bovine leukemia antibody test kit was purchased from Japan New Chisso Corp., Tokyo, Japan. Ferrozine was purchased from Sigma (St. Louis, MO, USA). Immuno Plate Maxisorp F96, Alkaline phosphatase (ALP)-conjugated NeutrAvidin, and EZ-link™ sulfo-NHS-biotin were purchased from Thermo Fisher Scientific (Waltham, MA, USA). Other reagents used were of analytical grade. Pure water (Elix water) was produced from tap water using an Elix Advantage Water Purification System (Millipore, Billerica, MA, USA).

### Blood and milk from BLV-infected dairy cows

Peripheral blood samples were collected from the jugular veins of dairy cows on dairy farms maintained in the Saitama prefecture of Japan. All cows including normal dairy cows more than 2 years old were reared to the Japanese feeding standard for dairy cattle but at different sampling date. Following recovery from centrifuged coagulated blood, serum was stored at −20°C in the presence of 0.1% sodium azide until analysis. Serum samples were assessed for the presence anti-BLV antibody by ELISA kit. ELISA titers (S/P values) were calculated as follows: Absorbances (at 405 nm; A_405_) were determined per the kit instructions and corrected by subtracting background (A_405_ for reaction wells not coated with antigen). Corrected A_405_ values for experimental samples were normalized against corrected A_405_ values for the positive control (hence S/P). BLV-infected cows were identified based on ELISA titers (S/P values) over 0.3. Milk samples also were collected from the respective cows, and were defatted by centrifugation prior to ELISA testing.

All experiments were conducted following the established guidelines for animal welfare and were approved by the Committee on the Ethics of Animal Experiments of Kitasato University (Permit Number: 14-027).

### Iron measurement

Serum and milk samples were diluted twofold with Elix water, and (for each sample) a 150-μL aliquot was mixed with an equal volume of a solution of a reducing and iron-dissolving reagent (2 M thioglycolate, 2 M HCl, and 10% (w/w) trichloroacetic acid). The mixture was kept at room temperature for 15 min followed by a centrifugation at 16,000 × *g* for 15 min. Each resulting supernatant (150 μL) was neutralized by addition of 150 μL of neutralizing reagent [30% (w/w) sodium acetate], followed by addition of 15 μL of 10 mM ferrozine solution as a chromogen reagent. After incubation for 30 min, the absorbance of the mixture in each well was measured using a spectrophotometer at a wavelength of 562 nm. Iron concentration was determined using the molar extinction coefficient (εmM = 27.9) of the ferrous ferrozine complex at 562 nm (24).

### Ferritin preparation and antibodies to ferritin

Bovine spleen was collected at slaughterhouse and frozen for the preparation of ferritin until use. Bovine spleen ferritin was purified from the frozen spleen according to the previously described method (25). Rabbit antiserum to bovine spleen ferritin was prepared and partially purified by affinity chromatography as described previously (25). A portion of the affinity-purified antibody to the bovine spleen ferritin was biotinylated with EZ-link™ sulfo-NHS-biotin according to manufacturer’s instructions.

#### Ferritin Measurement

Ferritin in serum and defatted milk samples was measured by sandwich ELISA according to a previously described method (22, 25). Briefly, anti-bovine spleen ferritin antibody (100 μL at 200 ng/mL) in phosphate buffered-saline (PBS: 150 mM NaCl, 20 mM sodium phosphate, pH 7.2) was added to each well of a microtiter plate. After wells were washed with PBS containing 0.5% Tween 20 (PBST), a 300-μL aliquot of ELISA buffer (PBS containing 0.1% Tween 20 and 0.1% gelatin, pH 7.2) was added to each well, and the plate was held at room temperature for 1 h to block non-specific adsorption of ferritin protein with gelatin. Following another wash with PBST, 100-μL aliquots of bovine spleen ferritin standards (0.156–10 ng/mL) in ELISA buffer containing 0.5 M (NH_4_)_2_SO_4_ were added, and the plate was incubated at 37°C for 2 h. Serum and milk samples were diluted 6- or 10-fold with the same buffer used to prepare the standard solution. The diluted milk sample was heat-treated at 60°C for 20 min followed by centrifugation at 14,000 × *g* for 15 min, and the resulting supernatant was used to determine the ferritin concentration as previously described (22). Aliquots (100 μL) of serum samples and heat-treated milk samples were pipetted to wells of the plate along with the ferritin standard series. After washing with PBST, 100 μL of biotinylated rabbit anti-bovine spleen ferritin antibody (125 ng/mL) diluted in ELISA buffer was added to each well, and the plate was incubated at 37°C for 1.5 h. After washing, 100 μL of ALP-conjugated NeutrAvidin (1 μg/mL) diluted in ELISA buffer was added to each well, and the plate was incubated at 37°C for 1.5 h. After washing, the ALP enzyme reaction was carried out using disodium *p-*nitrophenyl phosphate as previously described (25). The concentration of *p*-nitrophenol produced by the ALP reaction was determined by measuring the absorbance at 405 nm.

### Statistical analysis

All data obtained are presented as mean ± SD. The statistical significance of the differences in means between two groups was determined by Student’s *t*-test according to the distribution of data. A *P*-value <0.05 was considered significant for all tests.

## Results

### Effect of BLV infection on iron metabolism in dairy cows

**Figure 1:**
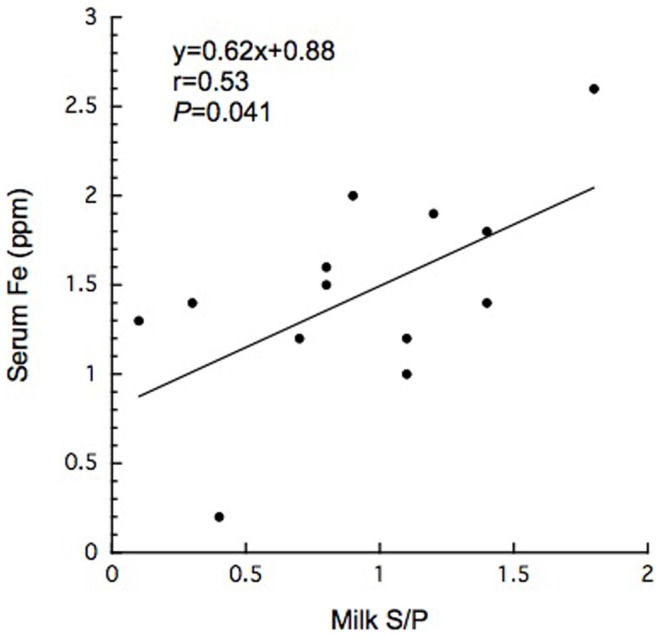
**Significant correlation between serum iron concentration and milk S/P value in BLV-infected dairy cows**.

**Figure 2:**
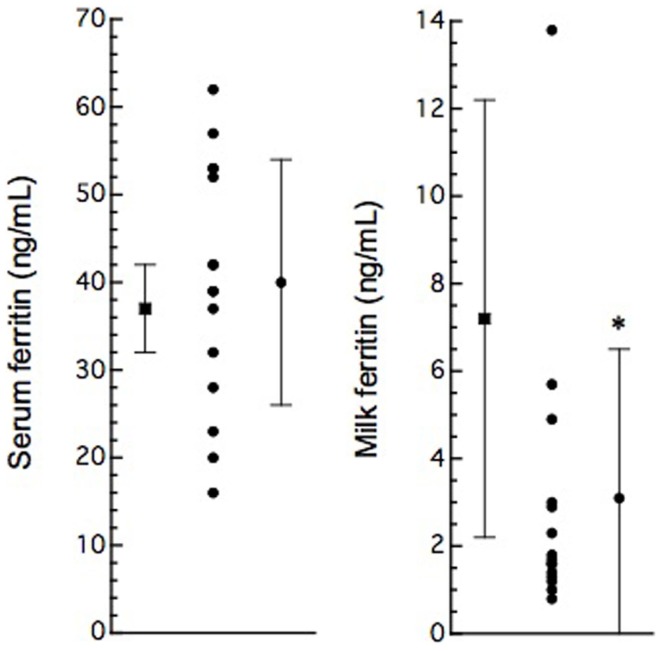
**Serum and milk ferritin concentrations in normal (solid boxes) and BLV-infected (solid circles) dairy cows**. Serum (*n* = 20) and milk (*n* = 15) ferritin concentrations are presented as mean ± SD from non-pregnant cows over 2 years old. Serum and milk ferritin concentrations are plotted as data from individual animals; vertical bars indicate mean ± SD (from Table 1). *: *P * < 0.05, compared to normal cows.

In this study, 15 BLV-infected dairy cows were selected according to the criteria of antibody titer against gp51 protein by ELISA kit. The antibody to gp51 antigen also was detected in the 13 milk samples, all but one of the cows that were BLV-positive by serum criteria. The antibody titers in the milk specimens were significantly lower (*P * < 0.001) than those in the sera of the BLV-positive cows (Table 1). The iron and ferritin concentrations in the milk of these cows also were significantly lower than the respective values in the sera (*P * < 0.01 and *P * < 0.001 in the iron and ferritin concentrations, respectively). The only significant correlation observed was that between serum iron concentration and milk S/P value (Figure 1). No significant difference was observed in serum ferritin concentrations between BLV-infected dairy cows (40 ± 14 ng/mL; *n* = 15) and normal dairy cows older than 2 years of age (37 ± 5 ng/mL; *n* = 20). By contrast, milk ferritin concentrations of BLV-infected cows (3.1 ± 3.4 ng/mL; *n* = 14) were significantly lower than those of normal dairy cows (7.2 ± 5.0 ng/mL; *n* = 17) (Table 1; Figure 2).

**Table 1:** **Age, anamnesis, and ferritin and iron concentrations, and S/P value in serum and milk of BLV-i nfected dairy cows**.

Sample no.	Age (year)	Anamnesis	Serum			Milk		
			S/P	Fe (ppm)	Ferritin (ng/mL)	S/P	Fe (ppm)	Ferritin (ng/mL)
1	2.6		1.5	1.4	16	0.3	0.14	1.7
2	2.8		1.7	1.3	54	0.1	0.07	4.9
3	3.5	Injury of parturient canal	2.2	1.6	37	0.8	0.39	1.6
4	3.7	Mastitis	2.7	2.6	23	1.8	0.07	0.8
5	3.8	Mastitis	2.3	1.4	42	1.4	0.35	5.7
6	4.0	Mastitis	2.0	1.9	53	1.2	0.12	13.8
7	4.0	Ketosis, pneumonia	2.8	1.2	42	0.7	0.10	1.3
8	4.2	Mastitis	2.4	1.2	28	1.1	0.24	2.9
9	4.8	Mastitis	2.3	1.2	32	1.1	0.14	1.8
10	4.9	Mastitis, dystocia	2.3	1.8	20	1.4	0.20	1.4
11	5.4	Abomasal displacement, pneumothorax	2.3	2.0	57	0.9	0.20	1.6
12	5.5	Mastitis, ketosis	2.3	1.4	39	NT	NT	1.2
13	5.7	Bronchitis	2.8	1.5	62	0.8	0.14	1.0
14	7.4	Dystocia, abomasal displacement	2.2	0.2	53	0.4	0.06	2.3
15	7.8	Mastitis	2.3	1.0	39	1.1	0.41	3.0
Mean ± SD	4.7 ± 1.5		2.3 ± 0.1	1.5 ± 0.5	40 ± 14	0.9 ± 0.5**	0.2 ± 0.1*	3.1 ± 3.4**

* and **: *P * < 0.01 and *P * < 0.001; significantly different from serum samples, respectively.

*NT: not tested*.

## Discussion

Bovine leukemia virus -infected cows often remain asymptomatic or aleukemic, presumably following establishment of proviral infection (1, 5, 6). Only one-third of infected cattle develop PL, a polyclonal expansion of B lymphocytes coexpressing CD5, high levels of surface immunoglobulin M (sIgM), and myeloid markers (1, 5–7). In the present study, antibodies to BLV antigen (gp51) were detected in all but one of the milk samples obtained from animals that were BLV-positive by serum titers. Notably, however, antibody titers in the milk of these cows were significantly lower than those in the respective sera. BLV particles may be found in the mammary glands of cows with subclinical mastitis (3, 15). Moreover, the presence of BLV proteins in the bovine milk exosome has been reported to correlate with the presence of anti-BLV antibodies in the milk (26). BLV-infected cows may release BLV particles or BLV proteins via the milk, providing a potential mechanism of BLV transmission. However, further studies will be needed to determine whether anti-BLV antibodies in the milk are sufficient to neutralize BLV particles and so prevent transmission via this biological fluid.

Bovine leukemia virus infection also has been shown to alter the expression of cytokines such as IL-2, IL-6, IL-10, and IL-12 (1). IL-6 may play a contributory role in viral latency due to the elevation of its level in blood circulation (1). IL-6 mediates hypoferremia of inflammation by the reduction of hepcidin, which in turn causes a decrease in intestinal iron absorption and the release of iron from macrophages (20). Most BLV-infected dairy cows exhibit past and present anamnesis, including inflammatory disease such as intrammamary disease. Although BLV infection may affect immune response to pathological agents due to immunosuppressive effects (1), it remains unclear how BLV provirus infection is associated with other pathological conditions. However, reports of an association between BLV infection and mastitis remain controversial (14, 15, 19). Previous reports indicated that zinc and iron levels in milk were significantly higher in California Mastitis Test-positive cows than in normal ones (27). BLV infection may activate cytokine receptor as well as B-cell growth cytokine (IL-4, IL-6, and IL-10) (11). On the other hand, in the comparison with previous report (28, 29), we failed to detect a significant difference in serum or milk iron concentrations when comparing between BLV-infected and normal dairy cows. Taken together, mammary gland is likely to affect systemic iron homeostasis for unknown reason. High ferritin levels were found in the sera of cows with leukemia (17). In humans, ferritin can be tumor marker and factors to monitor the effectiveness of treatment (23). However, it is unlikely to lead to the increase of serum ferritin when transformation does not occur. Interestingly, in the present study, milk ferritin levels were decreased in BLV-infected cows, even though the infected cows displayed inflammatory conditions (e.g., intramammary infection). This work also suggested the existence of a positive correlation between serum iron concentration and milk S/P value (Figure 1). Although the secretory mechanism of milk ferritin and iron in mammary gland remains to be resolved for individual viral stages, mammary gland infection by BLV appears to affect systemic iron homeostasis, seemingly independent of inflammatory condition, as described above.

Bovine leukemia virus infection can cause immune suppression in affected animals through multiple mechanisms (18). The results of this study suggest that BLV provirus infection also may disrupt iron metabolism. Further study will be needed to elucidate the relationship of iron metabolism between the whole body and the mammary gland.

## Conflict of Interest Statement

The authors declare that the research was conducted in the absence of any commercial or financial relationships that could be construed as a potential conflict of interest.
